# Amputation under the Influence of Ether

**Published:** 1847-11

**Authors:** 


*Amputation under the influence of Ether.*—We perceive by the Ithaca Daily Chronicle that Dr. J. E. Hawley, of Ithaca, formerly a resident of Buffalo, recently amputated a leg, the patient being under the influence of Ether, and entirely unconscious of pain.

